# Semi-automated image acquisition and analyses for broad users utilizing macro keyboards

**DOI:** 10.1093/jmicro/dfaf018

**Published:** 2025-04-03

**Authors:** Takaaki Watanabe, Toshiyasu Taniguchi

**Affiliations:** Department of Molecular Life Science, Tokai University School of Medicine, 143 Shimokasuya, Isehara, Kanagawa 259-1193, Japan; Department of Molecular Life Science, Tokai University School of Medicine, 143 Shimokasuya, Isehara, Kanagawa 259-1193, Japan

**Keywords:** macro keyboard, semi-automation, stream deck

## Abstract

Scientific research relies on microscopy. However, manual image acquisition and analysis are inefficient and susceptible to errors. Fully automated workflows are often task-specific, and current AI-based systems are costly and may face difficulties in new scenarios. Here, we introduce a semi-automated system utilizing macro keyboards to streamline workflows. Programming multi-action keys for tasks such as focusing, image capture and data analysis reduces the manual input, boosting efficiency and accuracy. This intuitive system saves time for both experienced users and trainees. This cost-effective solution improves accessibility, flexibility and usability, supporting not only diverse imaging applications but also broader scientific instrumentation processes.

## Teaser

This report presents a semi-automated microscopy system using macro keyboards to streamline workflows. Programming multi-action keys for tasks such as focusing, image capture, and data analysis reduces the manual input, boosting efficiency, and accuracy. This cost-effective solution improves accessibility and usability, supporting diverse imaging applications and broader scientific instrumentation processes.

Microscopy plays a crucial role in numerous scientific and industrial fields, including biological research and materials science. However, the acquisition and analysis of microscopic images often involve time-consuming manual processes that require repetitive manual inputs and adjustments [1]. These operations can result in significant performance constraints, particularly when handling extensive image collection or intricate analysis procedures. Fully automated workflows are often limited to specific tasks, and current AI-based systems are costly and may struggle with unfamiliar situations or fail to detect unexpected errors [2–4]. To address these challenges and improve efficiency and precision, semi-automated systems are gaining importance [5–10].

This study introduces a semi-automated approach for microscopic image acquisition and analysis that utilizes macro keyboards to streamline repetitive tasks. By programming macros to execute predefined command sequences, including microscope focusing, image capture, parameter adjustment and image analysis, this system reduces manual intervention while maintaining adaptability. Macro keyboards provide an affordable and accessible method to enhance these workflows without requiring specialized automation equipment. However, few macro keyboards have been used for capturing and analyzing microscopy images in research settings. The proposed system allows users to initiate complex, multistep processes with a single key press, thereby reducing human error, saving time and enhancing the overall efficiency of image acquisition and analysis. This approach is particularly advantageous in environments in which precise image acquisition is critical. However, full automation may be impractical owing to complexity or financial constraints [2–4]. This report outlines the design and implementation of the system, highlighting its applicability across diverse microscopy scenarios. Additionally, we assessed its performance in terms of time efficiency, accuracy and user-friendliness, demonstrating how semi-automation through macro keyboards can significantly improve microscopy workflows.

**Fig. 1.**
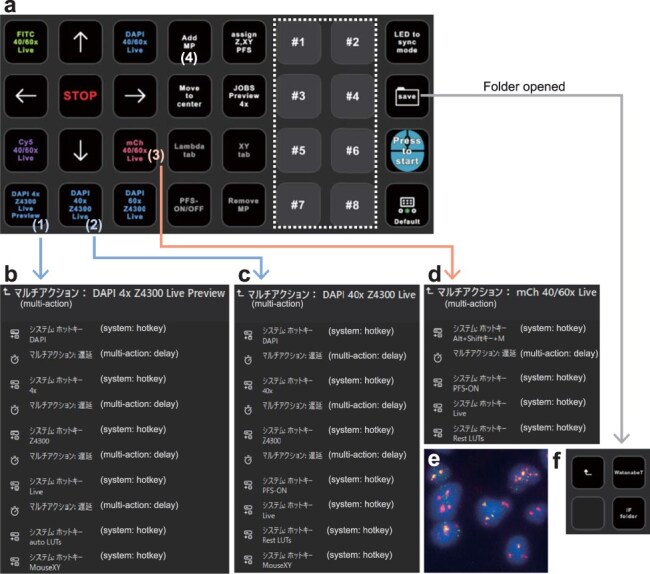
Semi-automated immunofluorescence imaging using Stream Deck XL. (a) Operational page for immunofluorescence imaging. (b–d) Detailed settings of multiple key actions. Custom macros were assigned to shortcuts as follows: selecting a 4× or 40× objective lens (4× or 40×) and DAPI or mCherry (Alt+Shift+M) channel, setting an approximate *Z*-coordinate (Z4300), displaying LIVE images, enabling auto or reset LUTs mode for brightness and contrast adjustment and activating the PFS for continuous focus monitoring and adjustment. These keys acquire multiple actions through the drag-and-drop of these macros (see Supplementary Fig. 1a). By appropriately inserting delayed actions, it becomes possible to proceed to the next action after the completion of the previous action, enabling the smooth progression of multiple actions. (e) A representative immunofluorescence image. (f) Keys on a data-saving page opened through the SAVE key in panel (a). The ‘username’ key opens the destination folder for data saving, and the ‘IF folder’ key creates a new folder with an automatically added date and prefix.

**Fig. 2.**
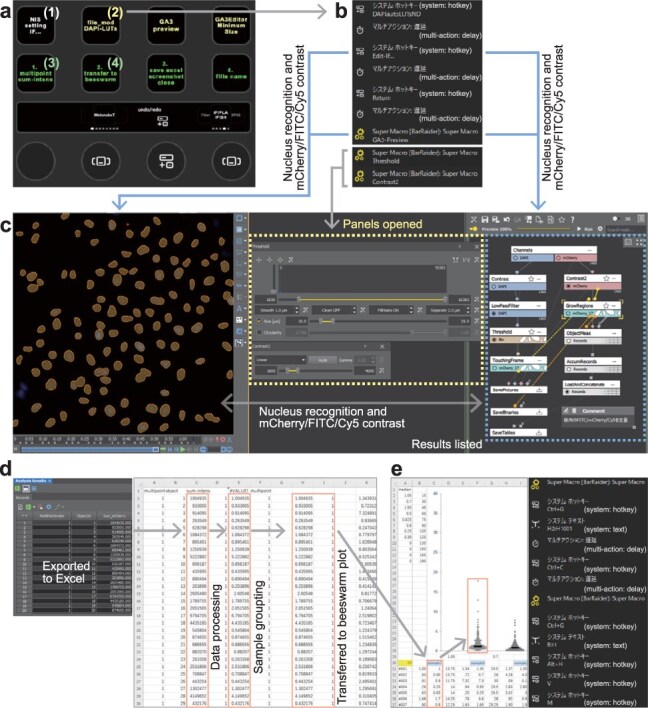
Semi-automated image analysis using Stream Deck Plus. (a) Operational page for immunofluorescence image analysis. The bottom four circles indicate action wheels, which can be assigned multiple simple tasks or repetitive actions. (b) Part of the detailed settings for the key labeled with ‘file_mod DAPI-LUTs’ in key (2) of panel (a). The sequences of macros can initiate an NIS-Elements module to adjust DAPI contrast for accurate nucleus recognition (DAPIautoLUTsND), list the sum-intensity of mCherry/FITC/Cy5 fluorescence within identified nuclei (Edit-IF… and GA3-Preview) and open panels for manual adjustment of thresholds and contrast. The SuperMacro plugin allows for mouse clicks at designated *XY* monitor coordinates for operations that cannot be assigned to shortcuts (see Supplementary Fig. 1b). (c) Representative display images during analysis. The recognized nuclei are colored in orange. The nuclear recognition and mCherry/FITC/Cy5 fluorescence dynamic range can be adjusted using the panels in a yellow-dotted rectangle. (d) A representative workflow of the image analysis triggered by the key labeled with ‘1. multipoint sum-intens’ in key (3) of panel (a). (e) A representative workflow (left) and the part of detailed settings (right) of the data transfer triggered by the key labeled with ‘2. transfer to beeswarm’ in panel (a), key (4), which transfers the values of fluorescence sum-intensity to another Excel file to create beeswarm plots.

**Fig. 3.**
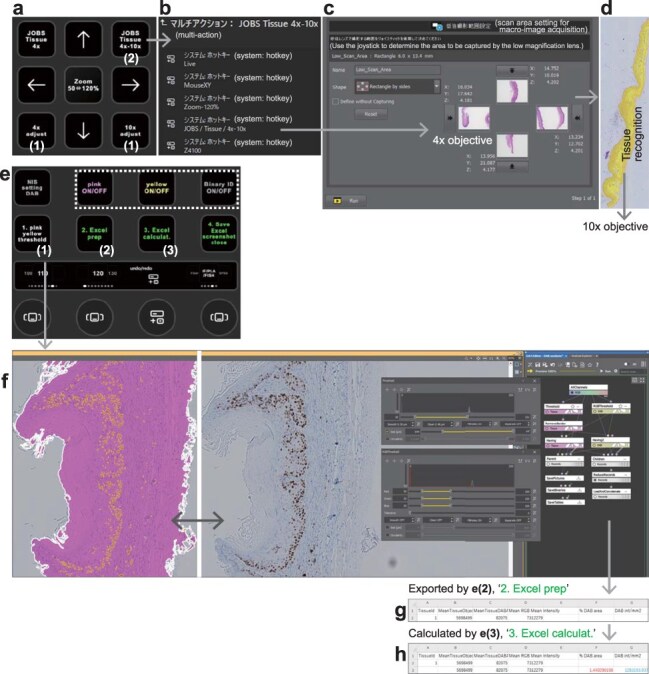
Semi-automated tissue imaging and analysis using Stream Decks. (a) Keys for tissue imaging. (b) Detailed setting of the multiple action key labeled with ‘JOBS Tissue 4×-10×’. JOBS is an automated acquisition pipeline tailored to user-specific projects. (c) Setting panel for designating the top, bottom, left and right of the 4× preview area. (d) A representative tissue that was recognized automatically. (e) Operational page for image analysis of DAB staining. The bottom circles indicate the action wheels, two of which are used to directly input the threshold values in the control panels shown in (f). (f) A representative display image during analysis. Recognition of the entire tissue section (pink) and DAB-stained areas (yellow) can be adjusted using the panels opened by the key labeled with ‘1. pink yellow threshold’ in panel (e). The keys toggle on or off individual binary IDs, binaries for all tissue sections and binaries for DAB-positive areas (Fig. 3e, white-dotted rectangle). (g, h) Representative of exported and calculated Excel sheets triggered by the keys labeled with ‘2. Excel prep’ and ‘3. Excel calculat.’ in panel (e), keys (2) and (3), respectively. This workflow is applicable to batch processes for the analysis of multiple tissue sections.

Operating microscopes involves navigating complex screens with numerous keys and context-dependent windows. This complexity makes it challenging for trainees to follow instruction manuals with screenshots, locate appropriate commands and understand the operational sequences. To address this issue, we employed Stream Deck XL, a versatile macro keyboard that features 32 customizable LCD keys capable of executing multiple actions with a single touch. This device allows key-function switching across various pages and folder structures. To enhance user experience, we engineered the system to execute all functions for each imaging process on a single screen with 32 keys. Here, we present a profile for the semi-automated immunofluorescence imaging of multidimensional samples composed of various treatments, cell types and time points (Fig. 1a; Supplementary Fig. 1). Our setup included a Nikon Ti2E microscope, Nikon Qi2 camera and Nikon D-LEDI for fluorescence excitation, all controlled by Nikon NIS-Elements software. After placing the ibidi µ-Slide 8 Well with stained samples, users could instantly move to the center of any desired well by selecting a well number, e.g. #1, preconfigured with *X*-, *Y*- and *Z*-coordinates (Fig. 1a, white-dotted rectangle). To identify regions with suitable cell numbers and densities using 4’, 6-diamidino-2-phenylindole (DAPI)-stained nuclei, a single key labeled with ‘DAPI 4× Z4300 Live Preview’ (Fig. 1a, (1)) performs multiple actions: selecting a 4× objective lens and DAPI channel, setting an approximate *Z*-coordinate, displaying LIVE images and enabling automatic Look-Up Tables (LUTs) mode for automatic brightness and contrast adjustment. These actions are visually listed in Fig. 1b. Another key labeled with ‘DAPI 40× Z4300 Live’ (Fig. 1a, (2)) facilitates focus adjustment and imaging position determination by selecting a 40× objective lens and DAPI channel, setting an approximate *Z*-coordinate, displaying live images and activating the perfect focus system (PFS) for continuous focus monitoring and adjustment. These actions are listed in Fig. 1c. Specific keys allow the examination of images across various channels, including FITC, Cy5 and mCherry (Fig. 1a). For example, the actions of a key labeled with ‘mCh 40/60× Live’ (Fig. 1a, (3)) are shown in Fig. 1d. The addition of multipoint (labeled with ‘add MP’, Fig. 1a, (4)) and directional move keys (→, ↑, ← and ↓) allows users to select multiple positions within each sample. After acquiring the multipoint images (Fig. 1e), users can directly open the destination folder and create a new folder with an automatically added date (Fig. 1f).

Ordinarily, the process begins with a visual check of the sample position, followed by a series of steps: selecting the DAPI channel, changing to a 4× objective, activating the excitation light, locating the focal plane, enhancing contrast and exploring the imaging region by dragging the mouse. Subsequently, the objective is changed to a higher magnification, such as 40×; the *X*-, *Y*- and *Z*-coordinates of the imaging site are determined; the PFS is engaged to maintain focus; and other fluorescence channels are examined. Multiple imaging locations were established for multipoint acquisition. These procedures require manual actions, such as verifying and navigating the imaging area and finding the focal plane, along with 10–20 mouse clicks. These steps are repeated for each sample compared. Stream Decks can automate most of these operations using ∼5–10 macro keys, reducing the time required to complete these tasks by 50–75% compared to traditional methods. Moreover, the use of systematically arranged keys with color coding and task descriptions makes it easier to remember the workflow, reducing the need for beginners to repeatedly refer to the manual. This system significantly reduced the image acquisition time for experienced researchers from 1–2 h to <30 min. Moreover, all 15 undergraduate students, from the first to fourth year, found the system intuitive and quickly mastered its use. By employing this system, users could reduce fluorescence sample fading and avoid operational errors, such as collisions between the objective lens and samples.

The Stream Deck series of devices can be used to quantify and examine microscopic images. We introduce a protocol for the semi-automated analysis of immunofluorescence images, where DAPI signals are employed to detect cell nuclei and calculate the sum-intensity of mCherry/FITC/Cy5 fluorescence within each nucleus. Initially, we used the FancyZones feature in Windows 11 to set up display areas for the NIS-Elements image analysis software (Nikon), Excel data processing and beeswarm plot creation, facilitating efficient data transfer between NIS-Elements and Excel files. A single key labeled with ‘NIS setting IF…’ (Fig. 2a, (1)) organizes the layout of the analysis panels in the NIS-Elements. Another key labeled with ‘file_mod DAPI-LUTs’ (Fig. 2a, (2)) duplicates and renames the data for processing, keeping the primary data, initiates an NIS-Elements module to adjust DAPI contrast for accurate nucleus recognition and lists the sum-intensity of mCherry/FITC/Cy5 fluorescence within the identified nuclei. These actions are listed in Fig. 2b and a representative display image during analysis is shown in Fig. 2c. Manual adjustment of the threshold and contrast settings for each channel were used to optimize nucleus recognition and the mCherry/FITC/Cy5 fluorescence dynamic range (Fig. 2c, yellow-dotted rectangle). The third key labeled with ‘1. multipoint sum-intens’ (Fig. 2a, (3)) exports quantification results to Excel, processes and modifies data and header rows, reorganizes data by the sample group and eliminates empty cells (Fig. 2d). The next key action labeled with ‘2. transfer to beeswarm’ (Fig. 2a, (4)) involves moving data from each sample group into an Excel template for beeswarm plot generation, removing blank cells and preparing data for statistical analysis using SPSS software (IBM) (Fig. 2e). Eliminating blank cells effectively prevents the unintentional entry of zero values when using Excel, since excessive columns exist to handle various amounts of data. While most microscope-specific software packages can export data to Excel, there have been few options for further processing of the exported data or smooth data transfer between multiple Excel files. Ordinarily, these processes require manual scrolling through extensive datasets and careful copying and moving of data across multiple files to prevent mistakes. In contrast, the system in this study enables automation of these tasks by establishing highly organized Excel templates for this system through extensive trial and error, thereby minimizing the potential for human error. Using this system, an experienced researcher reduced the analysis time from ∼2–3 h to <20 min. Moreover, all 15 undergraduate students, from the first to fourth year, found the system easy to comprehend and quickly mastered its use. The ability of rapid processing enabled multiple analyses with different parameter settings, thereby enhancing the overall precision of analytical results.

The proposed system is also applicable to tissue section imaging and analysis. After configuring the brightness and exposure time for 4× and 10× objective lenses using keys labeled with ‘4× adjust’ and ‘10× adjust’, respectively (Fig. 3a, (1)), a single key labeled with ‘JOBS Tissue 4×-10×’ (Fig. 3a, (2)) initiates several automated processes listed in Fig. 3b: 4× preview tile-imaging within the designated scan area (Fig. 3c), automatic recognition of the tissue section (Fig. 3d), 10× high-resolution large-image capture and storage in a specified folder. For 3,3′-diaminobenzidine (DAB) immunohistochemical staining quantification, Stream Deck Plus was used. The initial key labeled with ‘1. pink yellow threshold’ (Fig. 3e, (1)) launches a program that automatically detects the entire tissue section and the DAB-positive regions (Fig. 3f). The thresholds were manually adjusted using toggle keys (Fig. 3e, white-dotted rectangle) for individual binary IDs, binaries for entire tissue sections and binaries for DAB-positive areas (Fig. 3f). The second key labeled with ‘2. Excel prep’ (Fig. 3e, (2)) exports the quantification results to Excel, where the data and header rows were processed (Fig. 3g). The operator then visually inspected the results, deleted the noise-related binaries and used the third key to calculate the total tissue section and DAB-positive areas for each group in Excel (Fig. 3e, (3): ‘3. Excel calculat.’). This macro also determines the percentage of DAB-positive areas and staining intensity per unit area, highlighting the calculated values (Fig. 3h). The fourth key labeled with ‘4. Save Excel screenshot close’ handles file saving, renaming and screenshot capture to document quantification parameters (Fig. 3e). This approach enhances data reliability, experimental reproducibility and information accessibility. Using this method, 4× preview imaging takes ∼1 min per slide, and combined 4× preview and 10× high-resolution imaging required 3–5 min per slide. DAB staining quantification and analysis were completed in ∼5 min per slide. In addition, this method can be applied to analyze multiple tissue sections in a batch process. Software options for pathological analysis include HALO, Patholoscope (VisualDx) and Patholoscope (Applied Spectral Imaging), although these solutions are associated with substantial costs.

Stream Decks and other macro keyboards are commonly employed in streaming, video production and managing audio, camera and lighting equipment. However, their potential applications in microscopic control have not been explored or documented.

The Stream Deck lineup includes several models, such as Neo, MK2, XL and Plus, which differ in the number of keys and features. These devices are priced between $100 and $300. Stream Deck Mobile offers free access to six keys, with additional keys available through a $25 yearly subscription. These devices provide exceptional capabilities for microscopic image capture and analysis:

1. Concise function descriptions and intuitive visual icons can be displayed on each key, allowing users to operate without extensive reference to manuals.

2. Key color-coding facilitates the creation of controls that represent fluorescent channels or specific software.

3. Liquid Crystal Display (LCD) backlighting ensures usability in darkroom environments.

4. Custom macros for user-specific microscopes can be assigned to shortcuts, enabling easy configuration of multiple actions for all users (Supplementary Fig. 1a).

5. The SuperMacro plugin allows mouse clicks at designated X- and Y-coordinates on the monitor to perform operations that cannot be assigned to shortcuts (Supplementary Fig. 1b).

We configured macro keys based on the standard functions available in the NIS-Elements software (Nikon). Consequently, it is feasible to create similar macro keys for microscopes from other manufacturers. This study presents an innovative challenge in combining devices in an unprecedented manner. The benefits described earlier extend beyond microscopy to various PC-controlled analytical instruments, such as High-Performance Liquid Chromatography, mass spectrometers and next-generation sequencers, as well as tasks involving experimental data analysis or manuscript preparation using software such as Word, Excel, PowerPoint, Illustrator and Photoshop. While fully automated workflows are often limited to specific tasks and current AI-based systems are costly and may struggle with unfamiliar situations or fail to detect unexpected errors [2–4], the semi-automated system using the Stream Deck series described here offers numerous advantages. The system offers easy implementation, flexible adaptability and cost-effectiveness. This system provides significant benefits to microscope users, researchers across various scientific fields and particularly to students just starting to learn and research advisors, aiming to save time and improve outcomes.

## Supplementary Material

dfaf018_Supplementary_Data
