# Supplementary material for: Semi-automated image acquisition and analyses for broad users utilizing macro keyboards
Source: Microscopy (Oxf). 2025 Apr 3;74(6):437–42. doi: 10.1093/jmicro/dfaf018 (PMC12687464; doi:10.1093/jmicro/dfaf018)
Supplement: dfaf018_Supplementary_Data [file FigS1_revised3.pdf › suppl_data/FigS1_revised3.pdf]

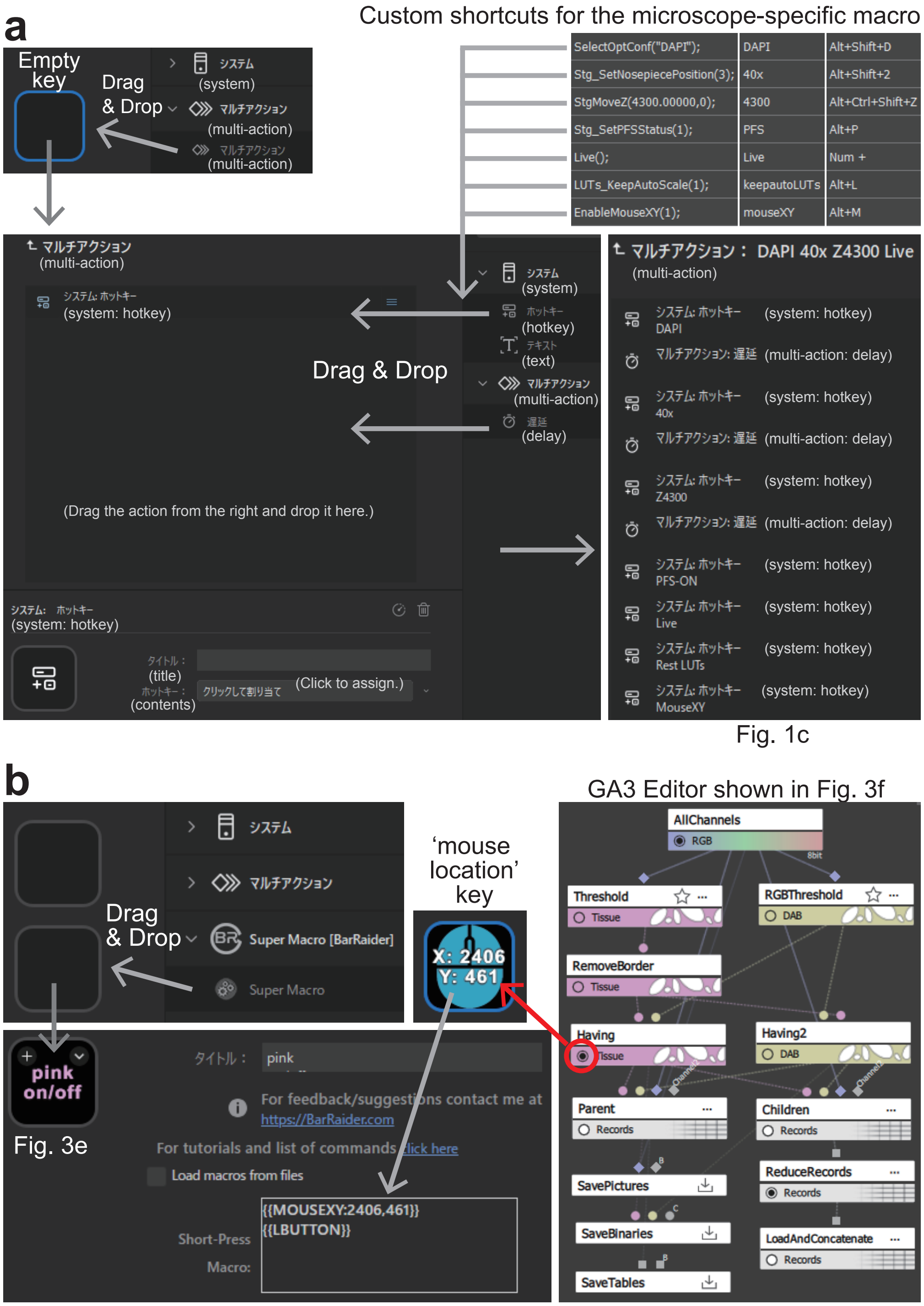
a
Custom shortcuts for the microscope-specific macro
SelectOptConf("DAPI"); DAPI Alt+Shift+D
Stg_SetNosepiecePosition(3); 40x Alt+Shift+2
StgMoveZ(4300.00000,0); 4300 Alt+Ctrl+Shift+Z
Stg_SetPFSStatus(1); PFS Alt+P
Live(); Live Num +
LUTs_KeepAutoScale(1); keepautoLUTs Alt+L
EnableMouseXY(1); mouseXY Alt+M
Empty key
Drag & Drop
システム (system)
マルチアクション (multi-action)
マルチアクション (multi-action)
システム: ホットキー (system: hotkey)
ホットキー (hotkey)
テキスト (text)
遅延 (delay)
Drag & Drop
(Drag the action from the right and drop it here.)
システム: ホットキー (system: hotkey)
タイトル: (title)
ホットキー: (contents)
クリックして割り当て (Click to assign.)
マルチアクション： DAPI 40x Z4300 Live
(multi-action)
システム: ホットキー DAPI (system: hotkey)
マルチアクション: 遅延 (multi-action: delay)
システム: ホットキー 40x (system: hotkey)
マルチアクション: 遅延 (multi-action: delay)
システム: ホットキー Z4300 (system: hotkey)
マルチアクション: 遅延 (multi-action: delay)
システム: ホットキー PFS-ON (system: hotkey)
システム: ホットキー Live (system: hotkey)
システム: ホットキー Rest LUTs (system: hotkey)
システム: ホットキー MouseXY (system: hotkey)
Fig. 1c
b
GA3 Editor shown in Fig. 3f
システム
マルチアクション
Super Macro [BarRaider]
Super Macro
Drag & Drop
'mouse location' key
X: 2406
Y: 461
pink on/off
Fig. 3e
タイトル： pink
For feedback/suggestions contact me at https://BarRaider.com
For tutorials and list of commands click here
Load macros from files
Short-Press Macro:
{{MOUSEXY:2406,461}}
{{LBUTTON}}
AllChannels
RGB
Threshold
Tissue
RGBThreshold
DAB
RemoveBorder
Tissue
Having
Tissue
Having2
DAB
Parent
Records
Children
Records
SavePictures
ReduceRecords
Records
SaveBinaries
LoadAndConcatenate
Records
SaveTables
